# Preparation of Fe_3_O_4_@PDA@Au@GO Composite as SERS Substrate and Its Application in the Enrichment and Detection for Phenanthrene

**DOI:** 10.3390/mi13010128

**Published:** 2022-01-14

**Authors:** Junyu Liu, Yiwei Liu, Yida Cao, Shihua Sang, Liang Guan, Yinyin Wang, Jian Wang

**Affiliations:** 1 College of Material and Chemistry & Chemical Engineering, Chengdu University of Technology, Chengdu 610059, China; liujunyu02022@163.com; 2 Department of Petroleum, Oil and Lubricants, Army Logistics Academy of PLA, Chongqing 401331, China; A439078225@163.com (Y.C.); avoidence@163.com (Y.W.); qwxywangjian@163.com (J.W.); 3 Department of Basic Courses, Army Logistics Academy of PLA, Chongqing 401331, China; liuvivien1210@gmail.com

**Keywords:** surface-enhanced Raman spectroscopy, nanocomposites, PAHs, enrichment detection

## Abstract

In this study, highly active Fe_3_O_4_@PDA@Au@GO surface-enhanced Raman spectroscopy (SERS) active substrate was synthesized for application in the enrichment and detection of trace polycyclic aromatic hydrocarbons (PAHs) in the environment. The morphology and structure were characterized by transmission electron microscopy (TEM), energy-dispersive spectroscopy (EDS), X-ray diffraction (XRD) and UV–visible absorption spectrum (UV–vis spectra). The effect of each component of Fe_3_O_4_@PDA@Au@GO nanocomposites on SERS was explored, and it was found that gold nanoparticles (Au NPs) are crucial to enhance the Raman signal based on the electromagnetic enhancement mechanism, and apart from enriching the PAHs through π–π interaction, graphene oxide (GO) also generates strong chemical enhancement of Raman signals, and polydopamine (PDA) can prevent Au from shedding and agglomeration. The existence of Fe3O4 aided the quick separation of substrate from the solutions, which greatly simplified the detection procedure and facilitated the reuse of the substrate. The SERS active substrate was used to detect phenanthrene in aqueous solution with a detection limit of 10^−7^ g/L (5.6 × 10^−10^ mol/L), which is much lower than that of ordinary Raman, it is promising for application in the enrichment and detection of trace PAHs.

## 1. Introduction

Containing a group of organic molecules comprised of two or more aromatic rings, polycyclic aromatic hydrocarbons (PAHs) are stable in structure and not easily degraded, thus they belong to the persistent hydrocarbons [1,2,3]. It is well-known that the more aromatic rings, the less easy it is to be degraded. PAHs are highly toxic with strong carcinogenicity, teratogenicity and mutagenicity, and 16 types of them appear in the environmental priority control list by the United States Environmental Protection Agency (EPA) according to their toxicity to human health [3,4,5]. Therefore, the development of trace detection for PAHs is significant. A rapid and sensitive method for PAHs’ detection urgently needs to be researched [6].

The current standard analytical methods for PAHs’ detection, including gas chromatography, gas chromatography–mass spectrometry (GC–MS) [7], immunoassay, electrochemical biosensing, and high-performance liquid chromatography (HPLC) [8,9,10,11,12,13] are currently broadly applied. These traditional detection methods have some disadvantages, such as having a time-consuming pretreatment process, and requiring the use of expensive, large-scale experimental instruments, which cannot meet the needs of rapid detection in the field.

As a powerful, rapid, nondestructive and sensitive analytical technique, SERS has been applied to on-site detection with a portable Raman spectrometer [14,15]. Raman spectroscopy has its unique advantages: no contact with the sample, no sample preparation, rapid analysis, its suitability for detecting liquid samples, and availability for high and low temperature and high pressure measurements in the environment. Compared with other analytical methods, it has remarkable advantages in environmental applications, including low sample pretreatment requirements, short analysis cycle, and on-site detection [16,17,18,19]. At present, the materials used to prepare the substrate are mainly nanomaterial precious metals (Au and Ag) and transition metals [20], as well as nonmetallic graphene. However, due to the poor adsorption capacity of the metal surface to PAHs, and hydrocarbons such as PAHs not having special functional groups that interact with the metal substrate, it is difficult to detect PAHs by using conventional SERS substrate. Therefore, surface functionalization of SERS substrate must be carried out. At present, one of the most commonly used method is to modify the plasmonic metal surface with different functional groups (e.g., cyclodextrin [21], alginate gel [22], viologen dications [23] and calixarene [24]), which can bring PAHs molecules close to the surface of nanostructured plasmonic metal. Although this method could achieve SERS detection of PAHs, the organic molecules used for modification are usually difficult to synthesize or expensive, which limits the practical application of SERS. Wang et al. prepared a Ag-nanoparticle–graphene hybrid for the direct detection of PAHs. The synthesis process is relatively simple, and the raw materials are not expensive, but the limit of detection for phenanthrene is 3.2 nM (~0.57 ppb) [25].

Until now, the combination of Fe_3_O_4_, polydopamine, GO and Au as a SERS substrate has not been reported.

Fe_3_O_4_ magnetic nanoparticles (MNPs) have many advantages, such as super paramagnetism, large specific surface area, fast adsorption rate, easy surface modification and good biocompatibility. They have a good separation and enrichment function in a complex matrix, and have been widely used in the biomedical, chemical catalysis, sewage treatment and biosensor fields [26,27]. In addition, they can be combined with SiO_2_, metal oxide, precious metal and graphene oxide to form core–shell structure composites, and will be widely applied in the future.

As an important neurotransmitter, dopamine is spontaneously oxidized and polymerized in air to form polydopamine (PDA) under weakly alkaline conditions. Polydopamine is a substance with rich functional groups, good hydrophilicity and excellent biocompatibility, which can cover many organic and inorganic materials, such as magnetic nanomaterial Fe_3_O_4_. It has a variety of applications such as drug loading, precious metal nanoparticle loading and organic pollutant adsorption [28].

Being an important derivative of graphene, graphene oxide (GO), also known as functional graphene, has a two-dimensional spatial structure and is a carbon-based nanomaterial with abundant hydroxyl, epoxy, carboxyl and other oxygen-containing energy groups. It has high specific surface energy, good hydrophilic and mechanical properties, and good adsorption to many chemicals. The accumulation and hydrophobic action of π-π between GO and PAHs can be used to give the PAHs a strong enrichment function [29,30].

Au NPs have unique optical properties, easy surface modification and excellent biocompatibility.So, functional Au NPs have a wide range of applications in many fields such as biosensing [31], detection of organic pollutants and heavy metal ions [32], and drug delivery [33].

Based on the above analysis of the advantages of the four materials, Fe_3_O_4_@PDA@Au@GO composite material was synthesized by assembly method to detect the low concentration of phenanthrene solution in the aqueous solution in this study. In this thesis, the effect of the mass of sodium citrate on the SERS was first analyzed. Then, the influence of different components of Fe_3_O_4_@PDA@Au@GO on the detection was analyzed and compared when the quaternary composite material was used as SERS substrate. This method was used to study the enrichment and detection effect of phenanthrene solution in aqueous solution and to obtain the detection limit, which provides new research methods and ideas for the synthesis of new composite SERS substrate and detection of trace PAHs in water.

## 2. Materials and Methods

### 2.1. Materials

Chloroauric acid tetrahydrate, sodium citrate, ferric chloride, anhydrous sodium acetate polyethylene glycol, dopamine hydrochloride, boron sodium hydrogen, phenanthrene and pyrene were purchased from McLean Biochemical Technology Co. Ltd. (Shanghai, China). Ethylene glycol, absolute ethanol and acetic acid were purchased from Chengdu Colon Chemicals Co. LTD (Chengdu, China). Graphene Oxide Dispersion was purchased from Sinopharm Nanjing Xianfeng Nanomaterials Technology Co. LTD (Nanjing, China). Trihydroxymethyl aminomethane was purchased from Shanghai Yuanye Biotechnology Co. LTD (Shanghai, China) [34]. All the above reagents were of analytical grade and can be used without further purification. The ultrapure water used in all experiments was obtained from a purification system [13].

### 2.2. Material Synthesis

#### 2.2.1. Synthesis of Au Colloid

Au colloid was synthesized by reducing chloroauric acid tetrahydrate (1.6 mM, 30 mL) with sodium citrate (0.022 g) as a reductant through the method of Freeman [13]. Firstly, 30 mL of chloroauric acid tetrahydrate (1.6 mM) was prepared and boiled under reflux with magnetic stirring. Secondly, 10 mL of trisodium citrate (0. 022 g) was added into the boiling solution of HAuCl_4_ and refluxed for another 30 min until it was changed from yellow to wine-red. Then, the solution was cooled to room temperature under stirring. Finally, the AuNPs were stored in a refrigerator at 4 °C for further use without further concentration or dilution [33,34,35].

#### 2.2.2. Synthesis of Fe_3_O_4_ Particles

Next, 0.45 g ferric chloride hexahydrate was dissolved in 14 mL ethylene glycol (EG), and the FeCl_3_·6H_2_O was completely dissolved by ultrasound for 20 min. Then, 1.2 g anhydrous sodium acetate (NaAc) and 0.1 g polyethylene glycol (PEG) were added to the FeCl_3_ solution, and stirred magnetically for 30 min. As polyethylene glycol (PEG) is difficult to dissolve, it was stirred for 30 min and ultrasonically stirred for another 15 min to obtain a uniform solution. The solution was transferred into a 50 mL high-pressure reactor, and gradually heated to 200 °C in an electric blast-drying oven for 8 h, then the kettle was taken out to cooled to room temperature. The Fe_3_O_4_ solution was poured into a beaker to separate the Fe_3_O_4_ nanoparticles from it by an external magnetic field, and the Fe_3_O_4_ nanoparticles were dried in a vacuum-drying oven at 60 °C for 6 h to obtain Fe_3_O_4_ magnetic nanoparticles after being washed alternately with ultrapure water and anhydrous ethanol 3~4 times [36,37,38].

#### 2.2.3. Synthesis of Fe_3_O_4_@PDA

First of all, 20 mg Fe_3_O_4_ magnetic nanoparticles were added into 60 mL anhydrous ethanol, and ultrasound was conducted for 10 min to allow 20 mgFe_3_O_4_ nanoparticles to completely disperse into 60 mL anhydrous ethanol. Then, 60 Ml Tris-HCl buffer containing DA-HCl (6.6 mmol/L) (dopamine hydrochloride) (pH = 8.5) was mixed with the above solution. After ultrasonication for 3 h, the as-obtained product was separated by an external magnetic field and the products were collected from the solution by magnetic separation method. Simultaneously, the Fe_3_O_4_ nanoparticles were covered by PDA and the state of Fe_3_O_4_@PDA became massive. The products were washed with ultrapure water 3 times and finally dried at 45 °C for 12 h [39].

#### 2.2.4. Synthesis of Fe_3_O_4_@PDA@Au

Au nanoparticles can load on the surface of Fe_3_O_4_@PDA nanoparticles by the classical electrostatic attraction method [39]. Firstly, 20 mg Fe_3_O_4_@PDA nanoparticles were added into 4 mL anhydrous ethanol and completely dispersed under ultrasonic dispersion for 20 min at room temperature. Then, the as-prepared Fe_3_O_4_@PDA solution was put into the solution of Au nanoparticles under mechanical stirring for 30 min at room temperature [28]. Finally, Fe_3_O_4_@PDA@Au nanocomposites were successfully prepared by coating Au nanoparticles (NPs) on Fe_3_O_4_@PDA.

#### 2.2.5. Synthesis of Fe_3_O_4_@PDA@Au@GO

Fe_3_O_4_@PDA@Au was washed three times and added into 2 mL ultrapure water [40]. The Fe_3_O_4_@PDA@Au aqueous solution was mixed with 0.5 mL (0.5 mg/mL) GO dispersion, adding an appropriate volume of deionized water to keep the total volume fixed to 12 mL. After being mechanically stirred for 3 h, Fe_3_O_4_@PDA@Au@GO was separated from the mixture by an external magnetic field [40]. Finally, the as-prepared product was washed with deionized water 3 times to obtain the Fe_3_O_4_@PDA@Au@GO product [28].

### 2.3. Characterization

X-ray diffraction (XRD) data were recorded on a D8-Advance diffractometer (Brouker, Germany) with Cu as the target source. Scans were made in the 2θ range of (5~85)° with a step size of 0.02° and a count time of 0.15 s per step [40]. The size, morphology and surface composition of Fe_3_O_4_@PDA@Au@GO were characterized by TEM (FEI Talos F200×). The UV–vis spectrum of AgNPs colloids was obtained on a Shanghai Meipuda Instrument Co. LTD UV-1600PC spectrophotometer [41]. The Raman and SERS spectra were obtained by a portable Raman spectrometer (Metage Opal 2800, Metage company, London, England) with an excitation wavelength of 785 nm [40]. The laser power and the integration time were set as 400 mW and 90 s, respectively [41].

### 2.4. Detection Method

We turned on the Metage portable spectrometer (785 nm excitation light) [42] to preheat it for 30 min, and checked the instrument and laser to ensure they were running normally. The PAHs solution and the prepared Fe_3_O_4_@PDA@Au@GO substrate were dropped into a brown bottle in proportion. The integral time was 10 s~50 s, and the laser intensity was 90. Each sample was collected 5 times, and the average value was taken. After deducting dark current, the Raman signal was detected in the order from high concentration to low concentration.

## 3. Results and Discussion

### 3.1. Materials Characterization

#### 3.1.1. UV–Vis Characterization

**Figure 1:**
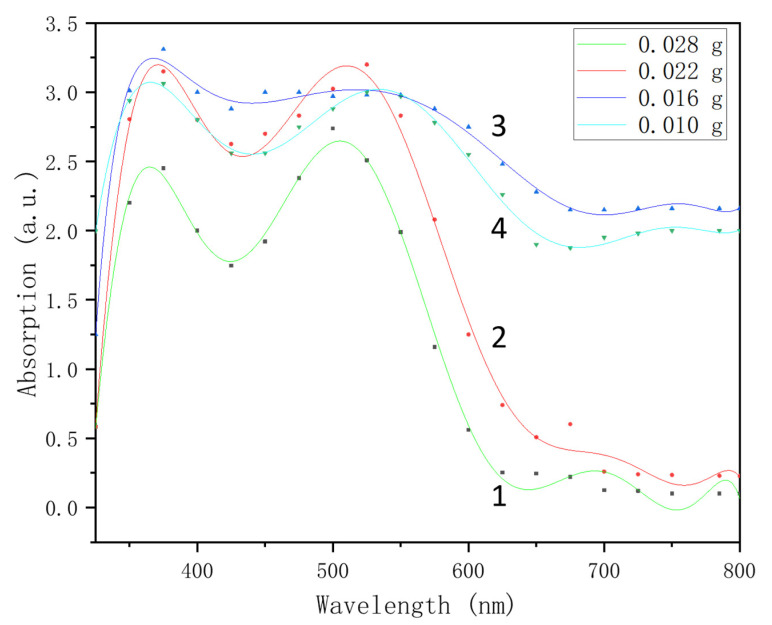
The UV–vis spectra of Au NPs synthesized by various masses of sodium citrate (1) 0.028 g; (2) 0.022 g; (3) 0.016 g; (4) 0.010 g.

The UV–vis spectra in Figure 1 demonstrate the maximum absorption of Au NPs synthesized by various masses of sodium citrate, which are located at the range of 350 nm–600 nm. The masses of sodium citrate were 0.028 g, 0.022 g, 0.016 g and 0.010 g, respectively. It can be seen from Figure 1 that when the mass of sodium citrate was 0.022 g, the absorption intensity at 385 nm and 525 nm were better. The SERS detection effect was the best when the mass of sodium citrate was 0.022 g, so the dosage of the reducing agent sodium citrate was 0.022 g [32].

#### 3.1.2. XRD Patterns of Fe_3_O_4_@PDA@Au@GO

**Figure 2:**
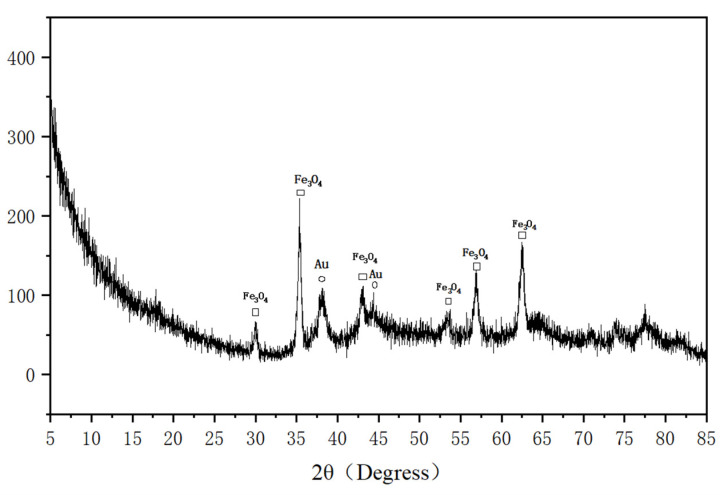
XRD patterns of Fe_3_O_4_@PDA@Au@GO.

In the X-ray diffraction (XRD) patterns of Fe_3_O_4_@PDA@Au@GO (Figure 2), six peaks appeared at 2θ = 30°, 35°, 43°, 53°, 57°, 62°, which are consistent with the XRD standard card PDF#79-0419 data [43]. The prepared Fe_3_O_4_ has very good structural characteristics and belongs to the cubic crystal system. Two major diffraction peaks (2θ = 38°, 44°) in the XRD pattern of the Au nanostars Fe_3_O_4_@PDA@Au@GO probe correspond to standard PDF#04-0784 and are indexed to the planes of Au in a cubic phase. The XRD pattern of the composite probe illustrates that the composites contain Fe_3_O_4_ and Au nanoparticles [43]. According to the above XRD analysis, there are Fe_3_O_4_ nanoparticles and Au nanoparticles in the Fe_3_O_4_@PDA@Au@GO SERS substrate.

#### 3.1.3. TEM Image of Fe_3_O_4_@PDA@Au@GO

**Figure 3:**
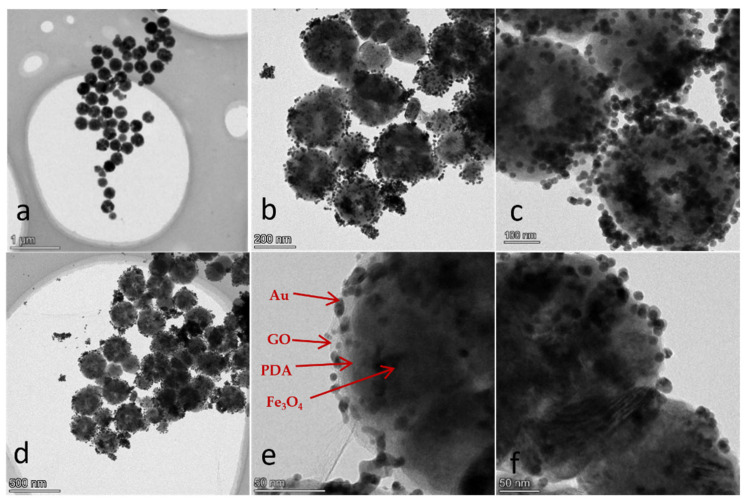
TEM image of Fe_3_O_4_@PDA/Au/GO nanocomposites. (**a**) transmission electron microscopy (TEM) image of 1 um; (**b**) transmission electron microscopy (TEM) image of 200 nm; (**c**) transmission electron microscopy (TEM) image of 100 nm; (**d**) transmission electron microscopy (TEM) image of 500 nm; (**e**) transmission electron microscopy (TEM) image of 50 nm; (**f**) transmission electron microscopy (TEM) image of 50 nm.

According to the corresponding TEM image of the Fe_3_O_4_@PDA@Au@GO presented in Figure 3, most nanoparticles are spherical in shape, with an average size of 1 μm. Further magnification at 100 nm and 200 nm shows that Au NPs are evenly distributed on the GO surface without large aggregates, indicating that Au NPs can be effectively adsorbed on the GO surface and Au NPs can be dispersed well by GO. As shown in the picture of amplification to 50 nm, Fe_3_O_4_ is in clusters, Au nanoparticles are the black dots, GO is coated in the outermost layer, and PDA is positioned in the interlayer in the center of the spherical Fe_3_O_4_ and Au nanoparticles.

**Figure 4:**
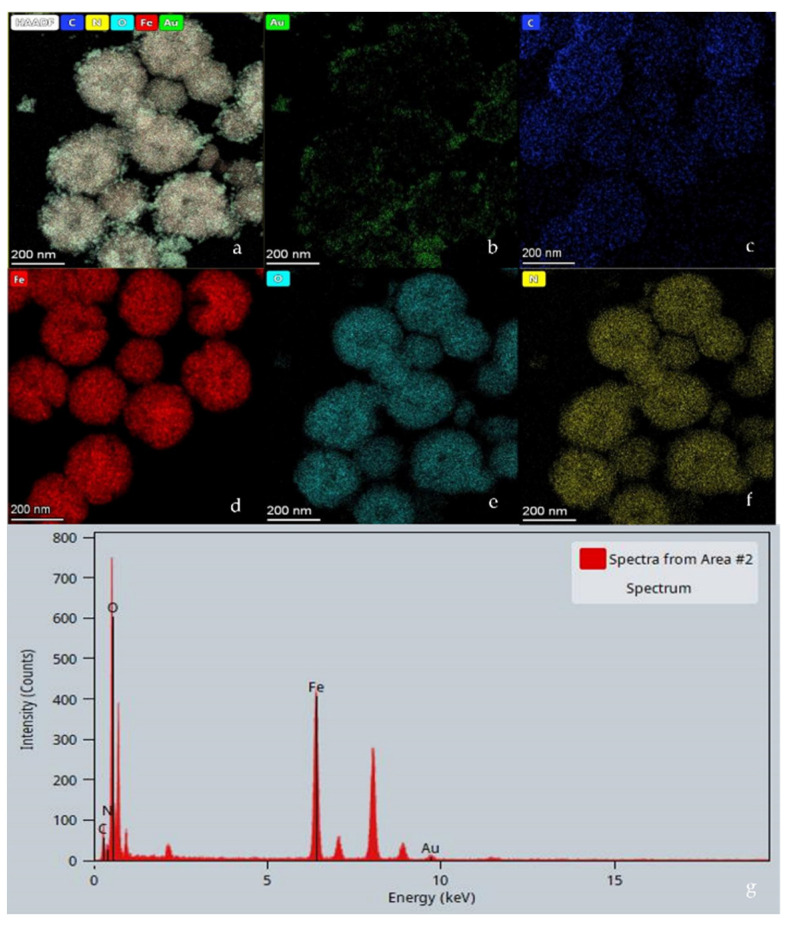
TEM mapping and EDS of Fe_3_O_4_@PDA/Au/GO nanocomposites. (**a**) the elemental mappings of Fe_3_O_4_@PDA/Au/GO; (**b**) the elemental mappings of Au; (**c**) the elemental mappings of C; (**d**) the elemental mappings of Fe; (**e**) the elemental mappings of O; (**f**) the elemental mappings of N; (**g**) EDS of Fe_3_O_4_@PDA/Au/GO.

As shown in Figure 4a,d, Fe is evenly distributed in the inside of the Fe_3_O_4_@PDA@Au@GO nanocomposite material, which proves that Fe_3_O_4_ acts as a magnetic substrate for SERS. In Figure 4b, Au is mainly distributed around the spherical substance. Figure 4c,e,f demonstrates that the distribution diameters of C, N and O elements are larger than Au, which indicates that GO is coated on the surface of Fe_3_O_4_@PDA@Au [13]. The EDS in Figure 4g accounts for the successful synthetization of Fe_3_O_4_@PDA@Au@GO.

### 3.2. Sample Preparation and SERS Measurement

Firstly, phenanthrene (0.1 g) was added to methyl alcohol as the solvent, shaken in ultrasonic for 30 min and stirred with magnetic stirring for 2 h. Secondly, the prepared materials were poured into a 100 mL volumetric flask and solutions of phenanthrene were prepared at a concentration of 1 g/L with methyl alcohol as the solvent. Lastly, a range of PAHs solutions at different concentrations were prepared by diluting them with methyl alcohol [13].

### 3.3. Impact of Various Masses of Sodium Citrate on SERS Detection Performance

**Figure 5:**
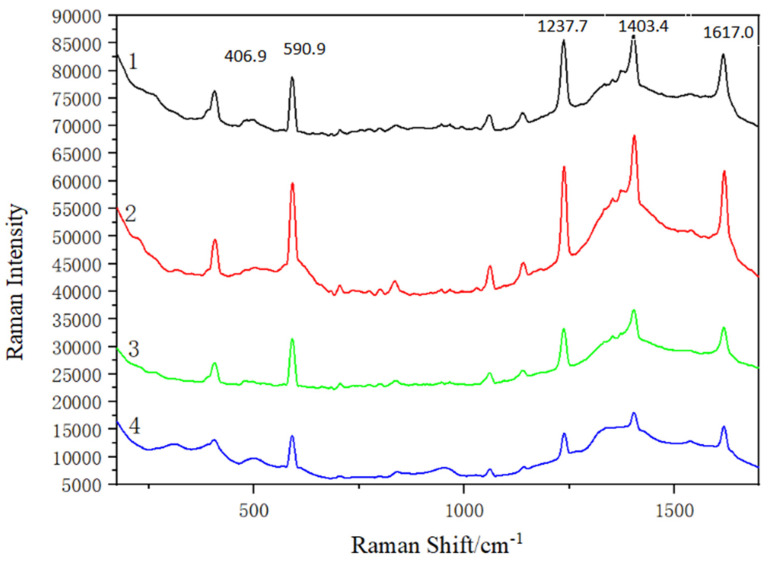
SERS signal of pyrene obtained on Au NPs substrate reduced by different masses of sodium citrate. (1) 0.028g; (2) 0.022g; (3) 0.016g; (4) 0.010g.

Figure 5 shows that Au NPs synthesized by various masses of sodium citrate were used as SERS substrates to investigate the effect of particle sizes on detection performance. The masses of sodium citrate were (1) 0.028 g (2) 0.022 g (3) 0.016 g (4) 0.010 g. The figure represents the SERS spectra of 10^−2^ g/L pyrene solution on Au NPs substrate by adding different amounts of sodium citrate, gold nanoparticles and pyrene mixed at the volume ratio of 1:3. Figure 5 shows that the peak intensity is the strongest in curve 2. Gold nanoparticles were prepared by sodium citrate solution configured with 0.022 g sodium citrate, and the detection effect of 10^−2^ g/L pyrene solution is the best, which is presented in Figure 5(2).

### 3.4. SERS Spectra of 10^−2^ g/L Phenanthrene Solution Detected by SERS Substrate

**Figure 6:**
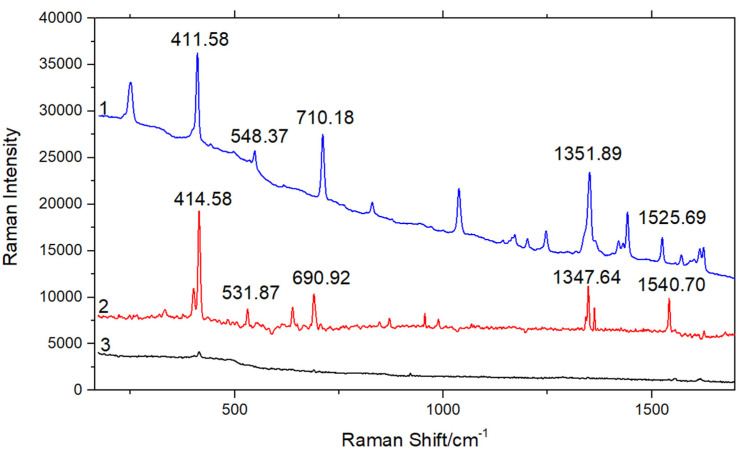
SERS spectra of phenanthrene. (1) phenanthrene solid; (2) 10^−2^ g/L phenanthrene SERS solution; (3) 10^−2^ g/L phenanthrene standard solution.

Phenanthrene solid, the mixture of gold substrate and phenanthrene solution (10^−2^ g/L) with volume ratio 1:1, and 10^−2^ g/L phenanthrene solution were added to three brown sampling bottles, and detected by a Metage Opal 2800 spectrometer. The detection results are displayed in Figure 6. The SERS spectra obtained by detection are shown in curves 1–3 in Figure 6. The comparison of curves 1 and 2 shows that the characteristic peak positions of phenanthrene solid were at 411.58 cm^−1^, 548.37 cm^−1^, 710.18 cm^−1^, 1351.89 cm^−1^ and 1525.69 cm^−1^, and the detected peaks of phenanthrene SERS solution were roughly the same. According to curve 3, the characteristic peak of 10^−2^ g/L phenanthrene standard solution almost disappeared. The characteristic peaks of phenanthrene can no longer be distinguished from ordinary Raman spectra. As can be seen from the above experimental results, Fe_3_O_4_@PDA@Au@GO substrate is crucial in SERS enhancement.

### 3.5. Effects of Each Component in Fe_3_O_4_@PDA@Au@GO on SERS Detection Performance

The purpose of this experiment is to detect the SERS activity of Fe_3_O_4_, Fe_3_O_4_@PDA, Fe_3_O_4_@PDA@Au, Fe_3_O_4_@PDA@Au@GO and the enhanced properties of various materials in Fe_3_O_4_@PDA@Au@GO substrate by preparing standard solution of 10^−2^ g/L and in accordance with the volume ratio of 1:1.

**Figure 7:**
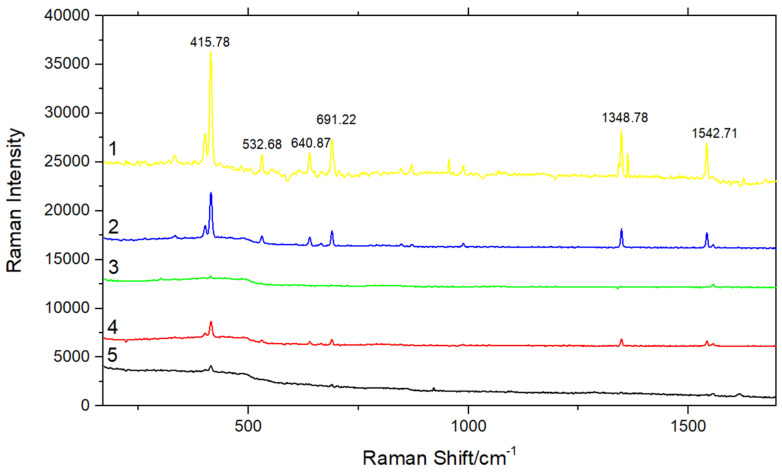
SERS signal of phenanthrene obtained on Fe_3_O_4_@PDA@Au@GO, Fe_3_O_4_@PDA@Au, Fe_3_O_4_@PDA and Fe_3_O_4_, respectively (1) Fe_3_O_4_@PDA@Au@GO substrate; (2) Fe_3_O_4_@PDA@Au substrate; (3) Fe_3_O_4_@PDA substrate; (4) Fe_3_O_4_ substrate; (5) 10^−2^ g/L phenanthrene solution.

It can be seen from curve 4 in Figure 7 that Fe_3_O_4_ has a certain enhancement effect on 10^−2^ g/L phenanthrene solution, but the effect is not obvious, while Fe_3_O_4_@PDA had no enhancement effect on the 10^−2^ g/L standard solution, and the characteristic peak is invisible from curve 3 in Figure 7, which might because PDA covered Fe_3_O_4_, meaning the SERS activity of Fe_3_O_4_ can not be shown. The characteristic peak positions of phenanthrene can be clearly seen from curve 2 at 415.78 cm^−1^, 532.68 cm^−1^, 640.87 cm^−1^, 691.22 cm^−1^, 1348.78 cm^−1^ and 1542.71 cm^−1^, which indicates that the SERS intensities of 10^−2^ g/L phenanthrene solution are increased by mixing with Fe_3_O_4_@PDA@Au substrate. It can be seen from curve 1 and 2 in Figure 7 that Fe_3_O_4_@PDA@Au enhanced the Raman signal of phenanthrene. When Fe_3_O_4_@PDA@Au@GO was used as the SERS substrate, the enhanced effect of the composite material was significantly better than that of Fe_3_O_4_@PDA@Au. This is because, on the one hand, GO can chemically enhance the Raman signal of phenanthrene, and on the other hand, GO can adsorb phenanthrene molecules through π–π interaction, narrowing the distance between phenanthrene and Au. Fe_3_O_4_@PDA@Au@GO substrate increases the adsorption capacity of 10^−2^ g/L phenanthrene solution by combining the electromagnetic enhancement of Au with the chemical enhancement of GO [29].

### 3.6. Influence of the Mixing Ratio of SERS Substrate and Phenanthrene Standard Solution on SERS Detection Performance

**Figure 8:**
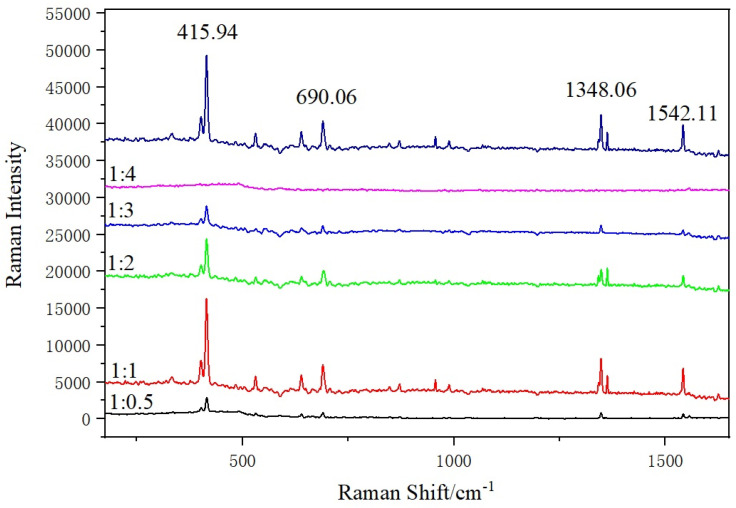
SERS spectra of different volume ratios of Fe_3_O_4_@PDA@Au@GO substrate and phenanthrene solution..

SERS substrate and 10^−2^ g/L phenanthrene solution were mixed by the volume ratios of 1:0.5, 1:1, 1:2, 1:3 and 1:4. The influence of the mixing ratio of SERS substrate and phenanthrene solution on SERS detection performance are shown in Figure 8.

The volume ratio of the substrates and phenanthrene might influence the SERS intensity. Its effect is researched by adding the SERS substrates into the phenanthrene solution (10^−2^ g/L) in different volume ratios of 1:0.5, 1:1, 1:2, 1:3 and 1:4, respectively [14]. There is a close linear correlation between the SERS intensities and the volume ratio. In Figure 8, it can be observed that the SERS intensity decreases when the volume ratio goes from 1:1 to 1:4, the peak disappears completely when the volume ratio is 1:4, which may be because the increase in the amount of the substrates promotes the adsorption of target molecules, whereas the SERS intensity is decreased when the volume ratio further goes from 1:1 to 1:0.5 [13]. Therefore, the volume ratio of 1:1 is selected for further experiments.

### 3.7. The Detection Limit of Phenanthrene Obtained on Fe_3_O_4_@PDA@Au@GO Nanocomposites SERS Substrate

**Figure 9:**
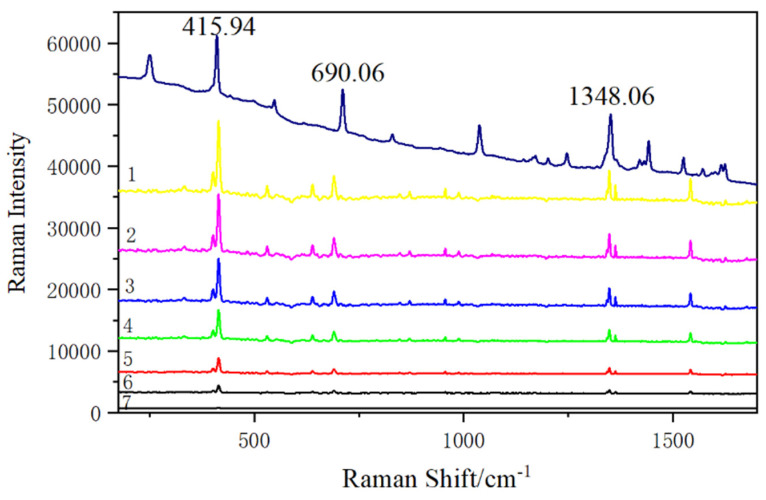
The detection limit of phenanthrene obtained on Fe_3_O_4_@PDA@Au@GO nanocomposite SERS substrate (phenanthrene solid. (1): 10^−2^ g/L phenanthrene SERS solution. (2): 10^−3^ g/L phenanthrene SERS solution. (3): 10^−4^ g/L phenanthrene SERS solution. (4): 10^−5^ g/L phenanthrene SERS solution. (5): 10^−6^ g/L phenanthrene SERS solution. (6): 10^−7^ g/L phenanthrene SERS solution. (7): 10^−8^ g/L phenanthrene SERS solution).

After mixing SERS gold substrate with phenanthrene of different concentrations at volume ratio 1:1, the spectra of different concentrations are detected. According to the above experimental results, the characteristic peak of phenanthrene is invisible in the ordinary Raman spectrum when the concentration of phenanthrene is 10^−2^ g/L. Therefore, the detection limit of phenanthrene by ordinary Raman is relatively high, and the detection of phenanthrene at low concentrations cannot be effectively carried out. The Raman signal of phenanthrene can be enhanced by the SERS substrate effectively. The detection limit of phenanthrene is explored by gradually reducing the concentration of phenanthrene solution, and the detected concentrations are 10^−2^ g/L, 10^−3^ g/L, 10^−4^ g/L, 10^−5^ g/L,10^−6^ g/L,10^−7^ g/L and 10^−8^ g/L, as displayed in Figure 9. Compared with the characteristic absorption peaks of phenanthrene solid in Figure 9, the characteristic absorption peaks of phenanthrene can be demonstrated in the SERS spectrum when the concentration of phenanthrene solution is 10^−2^ g/L, 10^−3^ g/L, 10^−4^ g/L, 10^−5^ g/L, 10^−6^ g/L, 10^−7^ g/L and 10^−8^ g/L. When the concentration is reduced to 10^−8^ g/L, the characteristic absorption peaks at all wavelengths are obviously weakened. However, the detection limit of phenanthrene by this method is 10^−8^ g/L, which is obviously lower than that of ordinary Raman since the GO in Fe_3_O_4_@PDA@Au@GO composite can adsorb and enrich phenanthrene in solution through π–π interaction, thus increasing the amount of phenanthrene on the composite. PDA prevents the shedding and reassembly of the Au, which enhances the electromagnetic properties of Au. The Raman signal is obviously enhanced through the electromagnetic enhancement effect of Au NPs and the chemical enhancement effect of GO [44]. This method can be further applied to the enrichment and detection of trace phenanthrene.

**Figure 10:**
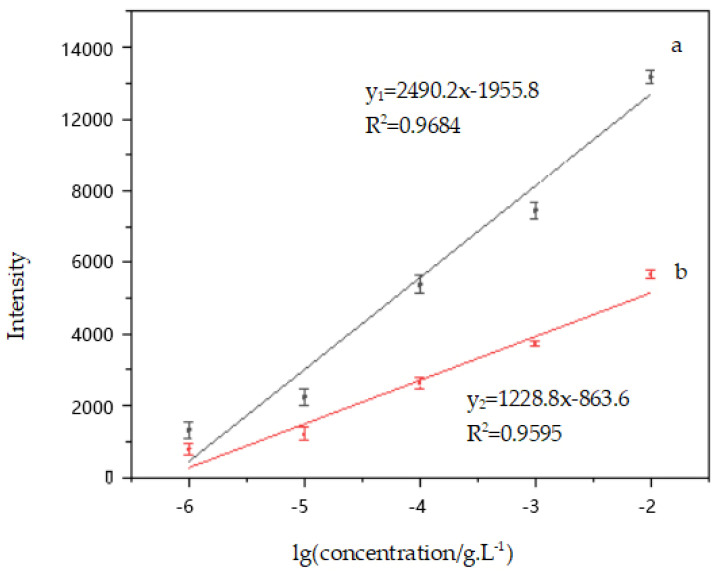
The linear correlations between the SERS intensity and the logarithm of phenanthrene concentration. (a) 415.24 cm^−1^; (b) 690.24 cm^−1^.

The SERS peak intensities versus the logarithm of phenanthrene concentration had a good linear relationship from 10^−6^ g/L to 10^−2^ g/L (Figure 10). The calibration equation can be described as (the characteristic absorption peaks located at 415.24 cm^−1^) y_1_ = 2490.2x − 1955.8 and the correlation coefficient (R^2^) is 0.9684. The linear regression equation (the characteristic absorption peaks located at 690.24 cm^−1^) is y_2_ = 1228.8x − 863.6 and the correlation coefficient (R^2^) is 0.9595. The error bars indicate the standard deviation derived from a total of 10 measurements. This shows that the substrate can be applied for rapid analysis and detection of phenanthrene at low concentrations.

### 3.8. Mechanism of PAHs–Substrates Interaction and Its Raman Enhancement

SERS measurements of PAHs detected on the SERS-developed substrates and Fe_3_O_4_@PDA@Au@GO can justify the hypothesis that the PAHs are bound to the substrates through the PAHs–Au–PDA–GO material interaction. The detection sensitivities of PAHs by this substrate can be enhanced by at least two orders of magnitude over that by Au NPs colloid [13]. According to the previous experimental results, the enhancement effect of the composite is not significantly increased after the addition of magnetic nanoparticles Fe_3_O_4_, indicating that Fe_3_O_4_ has no effect on the Raman signal. However, the presence of Fe_3_O_4_ can make the SERS substrate be quickly separated from the solution and can simplify the experimental operation. The results show that Au NPs are crucial to enhance the Raman signal based on the electromagnetic enhancement mechanism, and apart from enriching the PAHs through π–π interaction, GO also generates strong chemical enhancement of the Raman signal, and PDA can prevent Au shedding and agglomeration.

## 4. Conclusions

In this study, a layered self-assembly method was used to prepare Fe_3_O_4_@PDA@Au@GO substrate with high SERS activity, and the morphology and structure of the substrate were characterized by investigating the effect of different masses of reducing agent on the SERS detection. It was concluded that the optimal effect is seen with 0.022 g sodium citrate. Through the influence of different components of composite substrate on SERS detection performance, we also found that Au and GO enhanced the Raman signal in the Fe_3_O_4_@PDA@Au@GO substrate, and the existence of Fe_3_O_4_ can separate the substrate promptly and simplify the experimental steps. PDA has good adhesion and reactivity, and the composite nanoparticles have good magnetic response, which can quickly separate and enrich the molecules to be measured and shorten the pretreatment time. The detection limit is 10^−7^ g/L when Fe_3_O_4_@PDA@Au@GO is used to detect phenanthrene solution as the SERS substrate. Compared with the other SERS-based methods, the detection limit is obviously reduced. The applicability of the composite nanoparticles further proves that the SERS sensitivity is a simple, sensitive, environmentally friendly, and low sample pretreatment requirement technique which can be applied in the environmental detection of trace PAHs [45,46].

## Data Availability

The data presented in this study are available on request from the corresponding author.
